# Oral health behaviors and bacterial transmission from mother to child: an explorative study

**DOI:** 10.1186/s12903-015-0051-5

**Published:** 2015-07-03

**Authors:** Jorma I. Virtanen, Kimmo I. Vehkalahti, Miira M. Vehkalahti

**Affiliations:** Department of Community Dentistry, Faculty of Medicine, University of Oulu, FI-90014 Oulu, Finland; Medical Research Center, Oulu University Hospital, FI-90029 Oulu, Finland; Department of Social Research, Statistics, University of Helsinki, FI-00014 Helsinki, Finland; Institute of Dentistry, University of Helsinki, FI-00014 Helsinki, Finland

**Keywords:** Child, Health behavior, Mother, Multiple correspondence analysis, Oral health, Transmission

## Abstract

**Background:**

Health behaviors play a major role in the prevention of the most common oral diseases. To investigate health behaviors related to the potential transmission of oral bacteria from mother to child using novel multiple correspondence analysis (MCA).

**Methods:**

Mothers (n = 313) with children under three years attending two municipal child health clinics in Finland completed a self-administered questionnaire on health knowledge and behaviors such as sharing a spoon with their child, kissing on the lips, and the mothers’ tooth brushing, smoking, age, and level of education. We used MCA to reveal the relationships between the mothers’ behaviors and background factors, along with unconditional, binary, multivariable logistic regression models, odds ratios (OR) and their 95 % confidence intervals (95 %CI).

**Results:**

Of the mothers, 38 % kissed their child on the lips and 14 % shared a spoon with their child; 11 % believed that oral bacteria cannot be transmitted from mother to child. Two-thirds (68 %) of them reported tooth brushing twice daily, and 80 % were non-smokers. MCA revealed two diverging dimensions of the mothers’ behaviors: a ‘horizontal’ one showing clear evidence of relationships between tooth brushing, smoking, age and education, whereas the ‘vertical’ one revealed the mothers’ habits of kissing the child on the lips and sharing a spoon related to each other. Spoon sharing was related to the kissing on lips (OR 10.3), a higher level of education (OR 3.1), and, inversely, older age (OR 0.1), whereas kissing on lips behavior was inversely related to a higher level of education (OR 0.5).

**Conclusion:**

The study revealed two diverging dimensions of the mothers’ health behaviors. More emphasis in health education ought to be put to how to avoid bacterial transmission from caregiver to child during feeding.

## Background

Behaviors play a major role in the prevention of the most common oral diseases despite their infectious character. Behaviors related to the transmission of oral bacteria, together with diet and oral hygiene, are important in the etiology of dental caries in toddlers [1–3]. Consequently, protecting babies and toddlers from the maternal transmission of oral bacteria is considered vital to their oral health [2, 4]. The World Health Organization (WHO), for example, has published various guidelines and recommendations on health practices for mothers and caregivers [5, 6]. In addition, reducing the mother’s own oral bacteria is believed to minimize its transmission to the child and thus to decrease the risk for caries [7].

In many countries, children have limited or no access to dental care, although general health care services at nursery clinics, for instance, may be widely available. For this reason, the WHO has strongly advocated the integration of oral health care into general health care [8].

As part of public health service in Finland, maternity and child health clinics have for decades provided free-of-charge appointments to expecting mothers and children under school age. These services include general health checkups, vaccinations, and personal counseling [9], and the participation rate is nearly 100 % [10]. Children are scheduled for an average of three visits annually, but visit the clinic nearly every month during the first year of life [9, 11].

Since the 1970s, these services have included oral health care. Oral health education is available to parent groups in antenatal classes and as detailed instructions at individual appointments both during and after childbirth up to school-age [12]. In line with the recommendations of the WHO [8, 13], the topics address the importance of diet, smoking, oral hygiene and the use of fluorides. In addition, parents receive detailed instructions on how to avoid bacterial transmission from caregiver to child during feeding and when showing the child.

Our aim was to investigate the variety and complexity of health behaviors of the mothers of toddlers visiting child health clinics by use of multiple correspondence analysis method with respect to potential transmission of oral bacteria from mother to child.

## Methods

The study targeted mothers with children under three years attending public child health clinics in Finland. A self-administered anonymous questionnaire assessed mothers’ health behavior and background information. The Ethical Committee of Human Sciences at the University of Oulu approved the study.

The study population comprised of mothers with children under three years attending two municipal child health clinics in Southern Finland [14, 15]. The public health clinics with free-of-charge services were from two middle-sized towns (<50,000 inhabitants) with similar socio-economic and ethnic background; main source of livelihood in the towns is service trade and industry. A four to six month period was estimated to achieve a representative sample of mothers (ca. 330) from the health clinics. During the mothers’ routine visits to the clinics, health nurses distributed the questionnaires to all and collected them immediately after the mothers completed them. Nearly all invited mothers (>95 %) participated in the voluntary surveys and the final sample included 313 mothers [14, 15].

The question inquiring about the frequency of tooth brushing offered four answer options, later dichotomized to twice daily or less frequently. Based on the question “Do you smoke?”, the mothers were dichotomized as either smokers (daily, occasionally) or non-smokers (never).

Questions related to the potential transmission of oral bacteria from mother to child were as follows: “Do you share a spoon when feeding your child?”, “Do you kiss your child on the lips?”, “Do you clean the pacifier with your own mouth before returning it to your child?”; respondents answered each question with either “yes” or “no”. In addition, the mothers were asked to provide their opinion to the statement “Bacterial transmission from the mother’s mouth to the child’s mouth is impossible”; their answers ranged on a five-point Likert scale from total agreement to total disagreement, later dichotomized to those stating that such transmission is either possible or impossible.

The mothers’ background information included age in years (<25, 25-29, 30-34, 35-39, 40+), later categorized into four by combining the two oldest groups into one (35+). The mothers’ level of education was recorded as basic (compulsory = 9 years) education, vocational or professional education, or higher (polytechnic, university).

### Statistical methods

We applied multiple correspondence analysis (MCA) [16, 17] in order to explore and illustrate the relationships between the mothers’ health behaviors (tooth brushing and smoking), two of their health practices with their young children (sharing a spoon with their child and kissing the child on the lips), and the background variables (mother’s age and education level). MCA, an exploratory method often used to generate hypotheses, generalizes the simple correspondence analysis of frequency tables. MCA reveals the multidimensional structure inherent in the data based on pairwise frequency tables of the variables. The principal result of MCA is a graphical display called a biplot, most often a two-dimensional map of the categories and their relationships [16, 17]. In addition to the graphical displays, we produced a numerical summary of the MCA results describing the characteristics of the categories [18].

To further analyze the MCA findings, we used separate, unconditional, binary, multivariable logistic regression models: one of the health practices served as a dependent variable, while the other served as one of the explanatory variables along with the background variables. The results of the logistic regression analyses were presented as odds-ratios (OR) and their 95 % confidence intervals (95 %CI).

## Results

Of the mothers, 37 % were 30 to 34 year old and 40 % were below the age of 30. The majority of the mothers had attained a vocational education. Four (80 %) in five mothers were nonsmokers, 13 % reported smoking regularly, and 7 % occasionally. A total of 68 % of the mothers brushed their teeth twice daily (Table 1).

**Table 1:** Characteristics and behaviors of the mothers (n = 313) of toddlers visiting two Finnish child health clinics

Characteristics	Categories	%
**Age** (years)	<25	13
(Missing, n = 7)	25 to 29	27
	30 to 34	37
	35 +	23
**Level of education**	Basic	12
(Missing, n = 8)	Vocational	51
	Higher	37
**Smoking**	No	80
(Missing, n = 1)	Yes	20
**Tooth brushing**	2x/day	68
(Missing, n = 2)	<2x/day	32
**Kissing the child on the lips**	No	62
(Missing, n = 4)	Yes	38
**Sharing the spoon with the child**	No	86
(Missing, n = 5)	Yes	14
**Pacifier cleaned in mother’s mouth**	No	96
	Yes	4
**Bacterial transmission is possible**	No	11
(Missing, n = 21)	Yes	89

Of the mothers, 11 % believed that oral bacteria cannot be transmitted from mother to child. The most common health practices related to bacterial transmission from the mother’s mouth to the child’s mouth was kissing the child on the lips (38 %), followed by sharing a spoon when feeding the child (14 %). Virtually none of the mothers reported cleaning the child’s pacifier in their own mouth (Table 1).

**Fig. 1:**
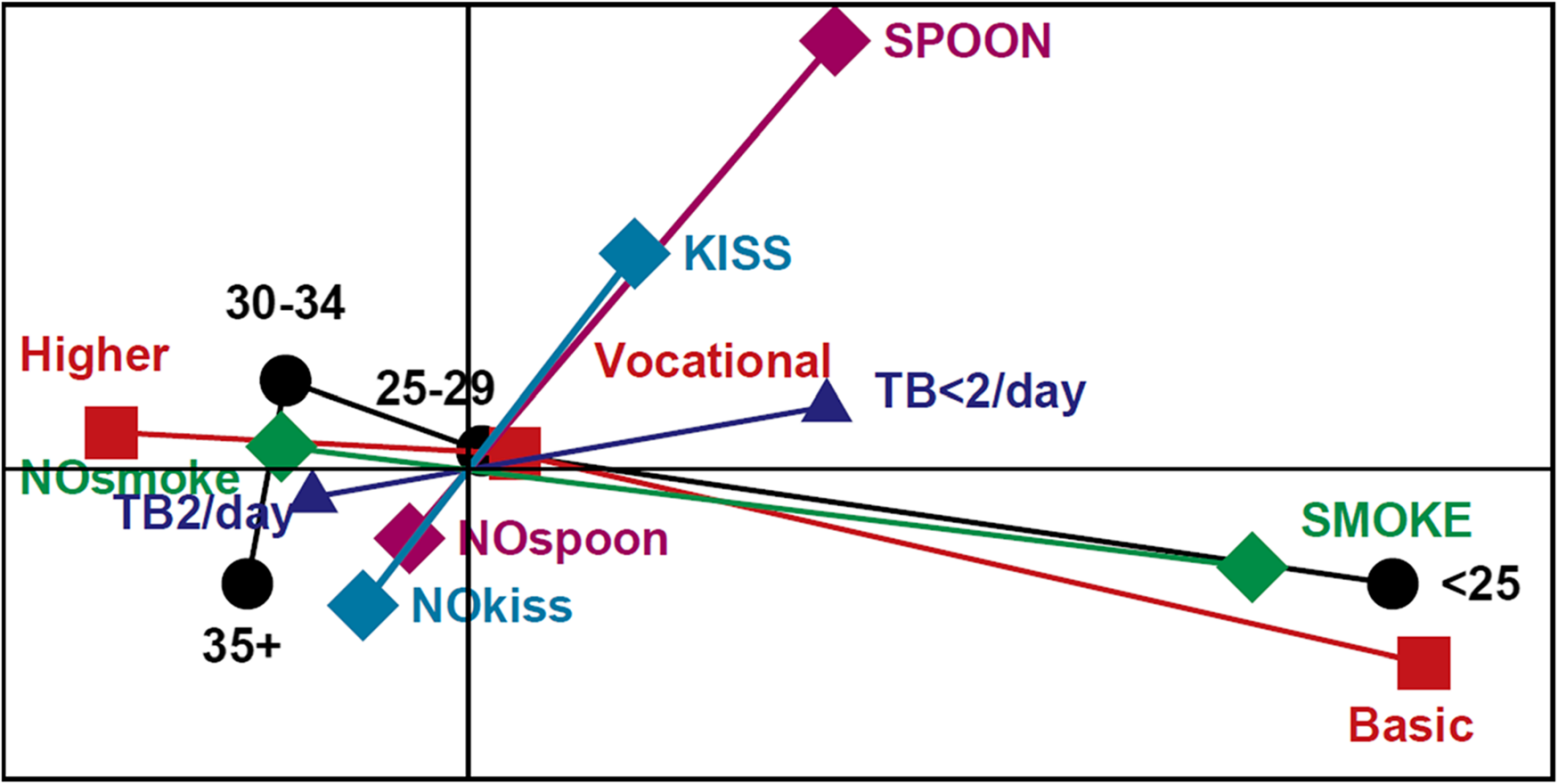
Variety and dimensions of dichotomized behaviors among mothers (n = 313) of toddlers visiting maternity clinics, visualized with multiple correspondence analysis. Black = age; Red = educational level; Green = smoking; Blue = tooth brushing; Turquoise = kissing the child on the lips; Violet = sharing a spoon with the child

According to Fig. 1, MCA revealed differences in mothers’ behavior related to potential transmission of bacteria as well as in tooth brushing and smoking habits. Unsurprisingly, certain categories of these variables, such as smoking, age under 25, and a basic level of education, appeared to be related to each other and were thus visibly close to each other in the graph forming the ‘horizontal’ dimension of behaviors. Brushing one’s teeth less than twice daily was to a certain degree related to these mothers’ health practices, as these categories were plotted on the graph in approximately the same horizontal direction. In addition, the health practices per se were clearly related: potential transmission of oral bacteria from mother to child by sharing a spoon with the child and kissing the child on the lips formed a clearly diverging ‘vertical’ behavioral dimension. These are visualized on the graph (Fig. 1) and they are supported by the detailed numerical summary presented in Table 2. For example, the points corresponding to the above mentioned behaviors had the greatest value of inertia (given in per mills of the total inertia of the data). The Coord columns present the coordinates of the points on horizontal and vertical dimensions of the graph *e.g.* sharing a spoon with the child (horizontal = 237 and vertical = 277) and not sharing a spoon (horizontal = -38 and vertical = -44), respectively. Absolute contribution (A-Cntr) shows the spoon sharing (54 and 336) and kissing (150 and 240) behaviors representing the greatest contributions of inertia on the vertical dimension.

**Table 2:** Detailed numerical summary of the multiple correspondence analysis by categories

Characteristics and Categories	Adjusted MCA model			Horizontal Dimension			Vertical dimension		
	Mass	Quality	Inertia	Coord	R-Cntr	A-Cntr	Coord	R-Cntr	A-Cntr
**Age**									
<25 yr	21	850	108	597	838	202	-74	13	21
25-29 yr	46	168	69	9	56	0	12	112	1
30-34 yr	64	829	62	-118	670	24	57	159	40
35+ yr	37	807	77	-142	636	20	-74	171	38
**Education**									
Higher	62	724	72	-231	716	91	24	8	7
Vocational	86	174	47	30	157	2	10	17	2
Basic	19	783	111	617	752	198	-125	31	57
**Tooth brushing**									
2/day	116	1027	32	-100	996	32	-18	31	7
<2/day	50	1027	74	231	996	74	41	31	16
Smoking									
No	135	821	25	-121	808	54	15	13	6
Yes	32	821	105	506	808	227	-63	13	25
**Sharing spoon**									
No	144	653	15	-38	276	6	-44	377	54
Yes	23	653	95	237	276	36	277	377	336
**Kissing on lips**									
No	103	655	42	-68	244	13	-87	410	150
Yes	64	655	67	108	244	21	140	410	240

Total inertia = 0.052854; of it the horizontal dimension explained 68.7 % and the vertical dimension 9.9 %

Mass = the mass of each point (x1000)

Quality = the quality of display in the solution subspace

Inertia = the inertia of the point (in per mills (‰) of the total inertia)

Coord = coordinate of the solution

R-Cntr = the (relative) contribution of the principal axis to the point inertia (x1000)

A-Cntr = the absolute contribution of the point to the inertia of the axis (in per mills (‰) of the principal inertia)

The first (horizontal) dimension explained 68.7 % of the total inertia (variation between the variables and their categories), while the second (vertical) dimension explained about 10 % of it. Additional dimensions explained less than 1 % each and hence had no practical significance.

For more detailed information, we used logistic regression models to analyze both health practices related to potential transmission of oral bacteria separately. The reference groups of the explanatory variables were selected similarly in both models. In the model for sharing the spoon with the child (Table 3), the most striking factors were kissing the child on the lips (OR 10.3; 95 %CI 4.3-24.4), a higher level of education (OR 3.1; 95 %CI 1.3-7.6), and, inversely, older age (OR 0.1; 95 %CI 0.03-0.6). Also, other age categories as well as a basic level of education had inverse but non-significant effects on this health practice. In the model for kissing the child on the lips (Table 4), the most striking factors were sharing the spoon with the child (OR 9.9; 95 %CI 4.2-23.5) and, inversely, a higher level of education (OR 0.5; 95 %CI 0.3-0.8).

**Table 3:** Factors explaining mothers’ habit of sharing their spoon with their child, as assessed by means of a logistic regression model on the mothers (n = 313) of toddlers visiting two Finnish child health clinics

Characteristics		OR	95 % CI
**Kissing the child on the lips**	No	ref.	
	Yes	**10.3**	**4.3 - 24.5**
**Current smoking**	No	ref.	
	Yes	1.4	0.5 - 4.1
**Twice-daily tooth brushing**	Yes	ref.	
	No	2.0	0.9 - 4.3
**Age** (years)	<25	ref.	
	25-29	0.3	0.1 - 4.5
	30-34	0.3	0.1 - 4.9
	35+	**0.1**	**0.03 - 0.6**
**Educational level**	Higher	**3.1**	**1.3 - 7.6**
	Vocational	ref.	
	Basic	0.7	0.2 - 3.0

Statistically significant values bolded

**Table 4:** Factors explaining mothers’ habit of kissing their child on the lips, as assessed by means of a logistic regression model on the mothers (n = 313) of toddlers visiting two Finnish child health clinics

Characteristics		OR	95% CI
**Sharing the spoon with the child**	No	ref.	
	Yes	**9.9**	**4.2 - 23.5**
**Current smoking**	No	ref.	
	Yes	1.1	0.5 - 2.4
**Twice-daily tooth brushing**	Yes	ref.	
	No	1.2	0.7 - 2.1
**Age** (years)	<25	ref.	
	25-29	1.8	0.7 - 4.5
	30-34	1.9	0.7 - 4.9
	35+	1.5	0.5 - 4.1
**Educational level**	Higher	**0.5**	**0.3 - 0.8**
	Vocational	ref.	
	Basic	0.8	0.3 - 2.0

Statistically significant values bolded

In further analyses to interpret the logistic regression models, the two models were visualized with separate MCAs (not shown in figures), one health practice at a time. The MCAs showed the different effects of higher education in the two models (OR 0.5 vs. OR 3.1) and their relationship to the mothers’ vocational education level, which served as the reference group in the logistic regression models. The mothers with a vocational education tended to kiss their child’s lips more often than did the mothers with a higher or a basic level of education. In contrast, the mothers with vocational education tended to share their spoon with their child less often than did the mothers with a higher level of education.

## Discussion

The study revealed two distinctive and significant dimensions of mothers’ health behaviors: one showed clear relationships between tooth brushing, smoking, age and education, and another revealed the mothers’ habits of kissing the child on the lips and sharing a spoon related to each other. Our study showed that MCA can be useful for illustrating the variety and complexity of the health behaviors of mothers and their practices related to potential bacterial transmission to their children.

### Complex behaviors

Health behavior entails a complex variety of knowledge, attitudes, and actions which positively or negatively impact health. Among the mothers, a minority believed that bacterial transmission from the mother’s mouth to the child’s mouth as a whole is impossible. However, only the “cleaning” of pacifiers in the mothers’ own mouth coincided with the mothers’ beliefs, whereas kissing the child on the lips was common probably reflecting the currently emphasized physical contact and shared pleasure in early mother–child interaction [19]. In line with previous reports [20–22], our findings of the mothers’ health behavior proved to be related to age and education level (see Fig. 1). Older age and higher level of education appeared to associate with optimal behaviors such as twice-daily tooth brushing and non-smoking. Surprisingly, mothers’ behaviors related to potential bacterial transmission by sharing a spoon with the child or kissing the child on the lips formed a clearly divergent behavioral dimension with weak or no relationship with age and level of education. Based on our study the two observed dimensions of health behavior appear divergent of each other.

Dental caries is a multi-factorial infectious and transmissible disease significantly influenced by health behavior. The mutans streptococci (MS) are the infectious agents most strongly associated with dental caries, and the significant reservoir from which children acquire these organisms is their mothers [4, 23]. Reducing amount of MS in highly MS-colonized mothers during the emergence of their children’s primary teeth at the age of about 6 to 12 months can prevent or delay colonization of the children by these bacteria for a prolonged period of time and decrease their risk for caries [7, 24].

### Finnish system

As part of public health services in Finland, a statewide network of maternity and child health clinics has been in operation since the 1940s and provides free services to all mothers and children under school age [9, 11]. The Ministry of Social Affairs and Health is responsible for guiding the development of maternity and child health clinics, whereas municipalities handle the practical arrangement of services. The high participation rates for these services, as well as systematic and frequent visits (totally 15-20 check-ups), enable the follow-up of mothers’ and their children’s health [9, 10].

### Multiple correspondence analysis (MCA)

MCA is a statistical method for studying the relationships of several categorical variables and is an exploratory method often used to generate hypotheses and in connection with logistic regression models or other methods of categorical data analysis [16–18]. This useful and versatile method has been applied in various fields of the health and medicine to social sciences, for example in detecting underlying structures in datasets, but has hardly ever served in dental research. The relationships between periodontal disease and social determinants of health were however, recently explored and visualized utilizing the method [25]. Our findings support the application of MCA to behavioral dental research as well as for supporting the interpretations of logistic regression analyses.

### Strengths and limitations

The use of MCA to illustrate the health behavior of mothers in connection with logistic regression analysis strengthens the findings of each. MCA was also used for supporting and clarifying the interpretations of findings from the logistic regression analysis. The exploratory findings can also be used to formulate hypotheses. The strength of MCA is its flexibility: distributional assumptions are unnecessary, and the relationships between the variables and their categories may be non-linear. The pair-wise analysis of the variables may be considered as a limitation of MCA, however the visualization of behaviors provides a major advantage [16, 17]. The graphical display of the relationships provides a user-friendly overview of the underlying relationships among the variable categories [26].

Questionnaire surveys have been broadly used to assess health related knowledge, attitudes or behavior of participants. The present study was based on mothers’ self-reported behavior and awareness when assessing potential bacterial transmission from mother to child. Even though self-reported outcome measures might be susceptible for socially desirable answering [27], it is unlikely the mothers would have overemphasized the mother–child interaction in question. The mothers came from two typical Finnish towns with similar socio-economic features and population structure. Since practically all mothers of toddlers visit the municipal child health clinics [10], our data well represent circumstances of Finnish mothers with small children. It is not likely that the observed two dimensions of mothers’ health behaviors differ considerably from the overall picture in the country. Further research is needed to confirm the transmission of microbes from mother to child by means of simple field-suitable tests, keeping in mind, however, the complexity of behaviors when new hypotheses are formed.

## Conclusion

The study revealed two diverging dimensions of the mothers’ health behaviors. Multiple correspondence analysis proved advantageous for illustrating the complex variety and dimensions of mothers’ health behaviors and health practices toward their toddlers. More emphasis in health education ought to be put to how to avoid bacterial transmission from caregiver to child during feeding.

## Abbreviations

CI: Confidence intervals
MCA: Multiple correspondence analysis
MS: Mutans streptococci
OR: Odds-ratio
WHO: World Health Organization
